# Association between parent and child physical activity: a systematic review

**DOI:** 10.1186/s12966-020-00966-z

**Published:** 2020-05-18

**Authors:** Therese Lockenwitz Petersen, Liselotte Bang Møller, Jan Christian Brønd, Randi Jepsen, Anders Grøntved

**Affiliations:** 1 Department of Sports Science and Clinical Biomechanics, Research Unit for Exercise Epidemiology, Centre of Research in Childhood Health, University of Southern Denmark, Campusvej 55, DK-5230 Odense M, Denmark; 2 Lolland-Falster Health Study, Centre for Epidemiological Research, Nykøbing F. Hospital, Fjordvej 15, 4800 Nykøbing F, Denmark; 3 University College Absalon, Region Zealand, Bispegade 5, 4800 Nykøbing F, Denmark; 4 Department of Physiotherapy and Occupational Therapy, Nykøbing F. Hospital, Fjordvej 15, Nykøbing F, 4800 Denmark

**Keywords:** Physical activity, Children, Parents, Family health, Accelerometer, Self-report

## Abstract

**Background:**

Childhood represents an important life stage for establishment of physical activity (PA) habits. Parents are assumed to play an important role in influencing children’s PA. Earlier reviews have mainly focused on parental modelling, encouragement, and support for PA, rather than the actual PA levels of parents. Therefore, the purpose of this review was to systematically summarize the evidence on the relationship between parent and child PA.

**Methods:**

Papers were identified using electronic databases and manual searches of reference lists. Papers reporting on associations between objectively measured child PA and at least one measure of parental PA were included. The quality of the papers was assessed using a modified version of the ROBINS-I tool. For interpretation of the results across studies, we produced albatross plots for all studies combined and by age-groups, sex of the parents, sex of the child, methodology of assessment of parental PA, and type of PA.

**Results:**

Thirty-nine papers were included with sample size of parent-child dyads ranging from 15 to 1267 (mean = 319 dyads, median = 227 dyads). The majority of studies were published from 2008 to 2018 and used accelerometry to assess PA. Most of the studies were classified as having moderate, serious, or critical risk of bias. The albatross plot for all studies combined showed that the clear majority of studies observed a positive relationship between parent and child PA. The plot suggested an average magnitude of correlation across studies to be around 0.13, and the overall impression was that this was fairly similar across child age-groups and gender of parent-child dyads. Studies using objective assessment of parental PA showed stronger relationship between parent and child PA compared with studies using self-report (average magnitude of correlation around 0.16 vs 0.04 respectively). No clear evidence was found for the strength of relationship being dependent on type of PA measure of parent and child (total PA, moderate-to-vigorous PA, steps), however, the relationship for light PA appeared weaker.

**Conclusion:**

This systematic review showed that the clear majority of studies observed a weak positive relationship between parent and child PA regardless of age of the child, the gender of the parent-child dyad, and type of PA.

**Trial registration:**

Registration in PROSPERO: CRD42019093462.

## Introduction

Physical inactivity has been identified as the fourth leading risk factor for global mortality by the World Health Organization (WHO) [1], and therefore, it has become an increasingly important topic in health promotion and health research [2]. Recent reports have estimated that globally, only one third of adults [2] and one third of children [3] reach the level of physical activity (PA) necessary to prevent health problems, as defined by the WHO [1]. The importance of regular engagement in PA is well-established in prevention of many non-communicable diseases [4] and for quality of life [5].

A complexity of physiological, psychosocial, familial, and environmental factors are potential determinants of PA behaviours in early life [6], and childhood represents an important life stage for establishment of PA behaviours, because these behaviours tend to track into adulthood [7]. In the family context, parents are assumed to play an important role in influencing children’s PA [8, 9]. Earlier reviews have summarised the evidence on associations between parental socioeconomic/sociodemographic factors, e.g. educational level, employment status, number of parents in the family [10], or parents’ self-reported behavioural and psychosocial support for child PA such as parental style, encouragement, and belief [8, 11, 12] and child PA. The results from these reviews have been mixed and inconclusive [8–12].

Many studies have relied purely on self- or parent-reported child PA rather than objective measures [8, 10, 11, 13], despite concerns over the reliability and validity [14]. Similar concerns have been raised when self-reports are used for determining general type, amount, intensity, and bout duration of PA among adults, however to a lesser degree [15, 16].

To further advance the understanding in this field, the purpose of this study was to systematically summarize the current research evidence on the relationship between PA levels of parents and children. Here, we define childhood as the age from 0 to 17 years.

## Method

This systematic review adheres to the PRISMA statement for systematic reviews (see Additional file 1) [17, 18] and is registered in PROSPERO (CRD42019093462).

### Literature search and search strategy

We searched the following electronic bibliographic databases for relevant studies: PubMed, EMBASE (from 1947-April 2018), PsycINFO, SPORTSDiscus, and The Cochrane Library (Cochrane Database of Systematic Reviews, Cochrane Central Register of Controlled Trials (CENTRAL), Cochrane Methodology Register) in March 2018.

A search strategy combining medical subject headings (MeSH and Emtree) and text words related to PA, parents, and children (Additional file 2) was developed by all authors from examining literature for common terminology utilized by published articles [11] and by consulting a health science librarian. The research terms were adapted to each specific database to ensure consistency of systematic searches (e.g. MeSH for PubMed and Emtree for Embase). Additional studies were identified through hand screening of reference lists of earlier reviews to ensure that no relevant articles were overlooked.

**Fig. 1:**
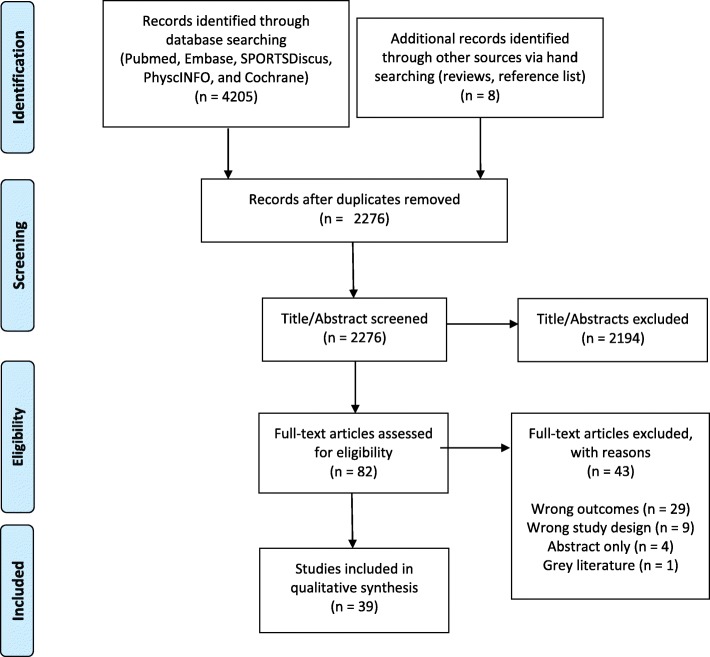
PRISMA flow diagram for the search and inclusion process for identification of articles

All potential references were imported into EndNote™ X8, and duplicates were removed. After removing the duplicates, all references were imported into Covidence (www.covidence.org) for further screening. The full screening protocol was repeated for all articles identified (Fig. 1).

### Eligibility for inclusion

Inclusion criteria were: 1) studies must report findings for at least one parent-child dyad. Included children should be between 0 and 17 years old. Parents could be biological or foster parents or any other legal guardians; 2) studies must report associations between parent and child PA. Child PA must be measured objectively using accelerometers or pedometers. Parent PA may be measured either objectively using accelerometers or pedometers or by self-report; 3) observational studies including cross-sectional data. Experimental studies were included if they reported cross-sectional data on the control and the intervention group; and 4) studies reported in English, Danish, Norwegian, or Swedish were read in their original languages.

### Article selection

The titles and/or abstracts of studies were screened independently by two authors (TLP and LBM) to identify studies that potentially met the inclusion criteria. A third author (RJ) made the final decision in case of conflicting results. If the title and/or the abstract suggested that a study was eligible, a full text copy of the article was obtained. Two authors (TLP and LBM) independently screened the full text of the included articles. Any disagreement over the eligibility of particular studies was resolved through discussion with a third author (RJ). Neither of the review authors were blinded to the journal titles.

### Data extraction

For the data extraction, we developed one tool for descriptive data and another tool for quality assessment. For descriptive data, we used a standardized, pre-piloted form, for which two authors (TLP and LBM) independently extracted data from the included studies (Table 1). The following data were extracted: author and year of publication, country of study, study design, age of children, family structure, measurement methods of parent and child PA, and reported associations for child PA. We also extracted objectively measured level of PA as 1) counts per epoch, and/or 2) an estimate of total time spent in moderate-to-vigorous physical activity (MVPA), and/or 3) steps per day.

**Table 1:** Descriptive data and assessment of risk of bias of studies reviewed

Author Year Country of study	Study design	Parent-child dyads, N	Age of children	Family structure	Measurement of PA^**a**^: children/parents	Reported or calculated p-value and direction (+/−) of association between children’s and parent’s PA	Overall risk of bias across domains
Moore et al. [19] - 1991 - USA	Longitudinal	99	4–7 y	Child and two biological parents	Caltrac Accelerometer/ Caltarc Accelerometer	Father-child total PA: *P* = 0.09 (+)	Serious
Sallis et al. [20] - 2002 - USA	Longitudinal	65	6–18 y	Non-specified	CSA model 7164 monitor/Questionnaire	Parent-daughter VPA^c^: *P* = 0.27 (+)	Critical
Loucaides et al. [21] - 2006 - Cyprus	Cross-sectional	36	10–12 y	One child and parents/guardians	Yamax digiwalker pedometer + questionnaire/Yamax digiwalker pedometer + questionnaire	Mother-child steps: *P* = 0.72 (−) Father-child steps *P* = 0.54 (+)	Serious
Oliver et al. [22] - 2010 - New Zealand	Cross-sectional	68	2–5 y	Child and primary caregivers. Triads from two-parent families. Dyads from single-parent families.	Actical accelerometer/ Actical accelerometer	Parent-child total PA: *P* = 0.01 (+)	Moderate
Fisher et al. [23] - 2010 - United Kingdom	Longitudinal	92	7–9 y M^b^ 8.31 ± 0.65 y	Children and parents	ActiGraph GT1M accelerometer/ Questionnaire	Parent-daughter MVPA^d^: *P* = 0.39 (+) Parent-daughter total PA: *P* = 0.37 (+) Parent-son MVPA: *P* = 0.33 (−) Parent-son total PA: P = 0.33 (−)	Critical
Heitzler et al. [24] - 2010 - USA	Cross-sectional	720	11–17 y M 14.7 ± 1.8 y	Youth and one parent/guardian	ActiGraph model 7164 accelerometer/The International Physical Activity Questionnaire	Parent-daughter MVPA: *P* = 0.06 (+)	Serious
Jago et al. [13] - 2010 - United Kingdom	Cross-sectional	139	6 y	Children and parents	ActiGraph accelerometer/ ActiGraph accelerometer	Parent-daughter MVPA: *P* = 0.598 (+) Parent-son MVPA: *P* = 0.47 (+)	Moderate
Fuemmeler et al. [25] - 2011 - USA	Cross-sectional	45	9–11 y M girls 10.6 ± 0.63 y M boys 10.6 ± 0.76 y	Child and mother and father	MTI ActiGraph accelerometer/ MTI ActiGraph accelerometer	Mother-child MVPA: *P* = 0.01 (+) Father-child MVPA: *P* = 0.02 (+)	Serious
Jacobi et al. [26] - 2011 - France	Cross-sectional	265	8–16 y	Children and father and/or mother	Yamax Digiwalker DW 450 Pedometer/Yamax Digiwalker DW 450 Pedometer + questionnaire	Mother-child steps: *P* = 0.00 (+) Father-child steps: P = 0.39 (+)	Serious
Ruiz et al. [27] - 2011 - USA	Cross-sectional	80	3–5 y M 4.2 ± 0.9 y	Children and parent/legal guardian	ActiGraph GT1M accelerometer/ActiGraph GT1M accelerometer	Parent-child MVPA: *P* = 0.0001 (+) Parent-child VPA: *P* = 0.56 (−)	Moderate
O’Dwyer et al. [28] - 2012 - Ireland/United Kingdom	Longitudinal	58	3–4.9 y M 3.7 y	Children and parents	ActiGraph GT1M accelerometer/ActiGraph GT1M accelerometer + a short version of Pre-PAQ questionnaire	Parent-child total PA: *P* = 0.00 (+)	Low
Craig et al. [29] - 2013 - Canada	Cross-sectional	283	5–19 y M girls 11.4 ± 0.5 y M boys 12.3 ± 0.4 y	Child and a responder 20 years of age or older who was identified as a parent or legal guardian	Pedometer (not specified)/Pedometer (not specified)	Mother-daughter steps: *P* = 0.03 (+) Mother-son steps: P = 0.00 (+) Father-daughter steps: P = 0.02 (+) Father-son steps: P = 0.05 (+)	Serious
Hnatiuk et al. [30] - 2013 - Australia	Longitudinal	153	3.5–18.7 months M T1: 3.5 ± 1.0 months M T2: 8.8 ± 1.0 months M T3: 18.7 ± 2.0 months	Child-parent dyads	ActiGraph GT1M accelerometer/The Active Australia Survey	Mother-child LPA^e^: P = 0.00 (−)	Serious
Hesketh et al. [31] - 2014 - United Kingdom	Cross-sectional	554	4 y M 4.1 ± 0.1 y	Mother and child	Actiheart monitor/Actiheart monitor + questionnaire	Mother-child LPA: *P* = 0.00 (+)	Moderate
Lloyd et al. [32] - 2014 - Australia	Cross-sectional	70	5–12 yM 8.4 ± 2.4 y	Oldest child and mother and/or father	Yamax 200 pedometer/ Yamax 200 pedometer	Father-child steps: *P* = 0.07 (+)	Serious
Jago et al. [33] - 2014 - United Kingdom	Cross-sectional	651	5–6 y M girls 6.0 ± 0.42 y M boys 6.0 ± 0.41 y	Children and at least one parent/caregiver	ActiGraph GT3X accelerometer/ActiGraph GT3X accelerometer	Mother-child MVPA: *P* = 0.01 (+) Father-child MVPA: *P* = 0.07 (+)	Moderate
Sigmundová et al. [34] - 2014 - Czech Republic	Cross-sectional	245	9–12 y M girls 10.44 ± 1.33 y M boys 10.57 ± 1.26 y	Children and parents	Yamax pedometer/Yamax pedometer	Parent-child steps: *P* = 0.00 (+)	Serious
Duncan et al. [35] - 2015 - USA	Cross-sectional	372	10–14 y M 12.06 ± 1.69 y	Families with a girl	ActiGraph GT3X+ accelerometer/Behavioral Risk Factor Surveillance System	Parent-daughter MVPA: (African) *P* = 0.00 (+) (Latino) *P* = 0.28 (+) (White) *P* = 0.28 (+)	Critical
Tu et al. [36] - 2015 - Canada	Cross-sectional	98	11–16 y M 13.1 ± 1.8 y	One child and one parent	ActiGraph GT3X/GT3X+ accelerometer/ActiGraph GT3X/GT3X+ accelerometer	Father-child MVPA: *P* = 0.00 (+)	Moderate
Tate et al. [37] - 2015 - USA	Cross sectional	493	8–14 y M 11.33 ± 1.49 y	One child and one parent	ActiGraph GT2M accelerometer/ActiGraph GT2M accelerometer	Parent-child MVPA: *P* = 0.00 (+)	Critical
Sijtsma et al. [38] - 2015 - Netherlands	Cross-sectional	230	2–5 y M 3.7 ± 0.6 y	Children and parents	Tracmor_D_ accelerometer/SQUASH questionnaire	Mother-child total PA: *P* = 0.14 (+) Mother-child MPA: *P* = 0.40 (+) Father-child total PA: *P* = 0.32 (+) Father-child MPA: *P* = 0.40 (+)	Moderate
Sigmund et al. [39] - 2015 - Czech Republic	Cross-sectional	245	9–12 y M girls 10.44 ± 1.33 y M boys 10.57 ± 1.26 y	Children and parents	Yamax pedometer/Yamax pedometer	Mother-daughter steps: *P* = 0.00 (+) Mother-son steps: *P* = 0.00 (+) Father-daughter steps: *P* = 0.00 (+) Father-son steps: *P* = 0.00 (+)	Serious
Hnatiuk et al. [40] - 2016 - Australia	Cross-sectional	127	3–4 y	One child and one parent	Actiheart monitors/Questionnaire	Mother-child MVPA: P = 0.37 (−) Mother-child LPA: *P* = 0.01 (+)	Critical
Abbott et al. [41] - 2016 - Australia	Cross-sectional	450	3–5 y M T1: 4.6 y M T2: 7.6 y	Children and both parents	ActiGraph GT1M accelerometer/ The Active Australia Survey	Mother-daughter MVPA: *P* = 0.29 (+) Mother-son MVPA: *P* = 0.05 (+) Father-daughter MVPA: *P* = 0.79 (+) Father-son MVPA: *P* = 0.85 (−)	Serious
Stearns et al. [42] - 2016 - Canada	Cross-sectional	440	7–8 y	Children and parents	Unsealed Steps Count (SC)-T2 pedometer/ Unsealed Steps Count (SC)-T2 pedometer + The Godin Leisure Time Exercise Questionnaire	Parent-child steps: *P* = 0.36 (+)	Serious
McMurray et al. [43] - 2016 - USA	Cross-sectional	199	7–10 y M 9.0 ± 0.9 y	One child and one parent/legal guardian	Actical accelerometer/ Actical accelerometer	Father-child MVPA: *P* = 0.009 (+)	Moderate
Solomon-Moore et al. [44] - 2017 - United Kingdom	Cross-sectional	1067	5–6 y M 6.01 ± 0.42 y	Children and parents	ActiGraph GT3X accelerometer/ ActiGraph GT3X accelerometer	Parent-child total PA: *P* = 0.005 (+)	Serious
Garriguet et al. [45] - 2017 - Canada	Cross-sectional	1328	6–11 y M 8 y	Biological parent-child dyad	Actical accelerometer/Actical accelerometer + The Canadian Health Survey	Parent-daughter MVPA: *P* = 0.00 (+) Parent-son MVPA: *P* = 0.10 (+)	Moderate
Brown et al. [46] - 2017 - United Kingdom	Longitudinal	406	9–10 y M 10.2 ± 0.3 y M follow-up 14.3 ± 0.3 y	Child and parents	ActiGraph GT1M accelerometer/ ActiGraph GT1M accelerometer	Father-child total PA: *P* = 0.30 (−)	Moderate
Song et al. [47]- 2017 - USA	Cross-sectional	55	3–5.99 y 10–12.99 y	Children and mothers	ActiGraph wGT3X accelerometer/ActiGraph wGT3X accelerometer + The Canadian Health Survey	Mother-child MVPA: P = 0.01 (+)	Moderate
Chiarlitti et al. [48] - 2017 - Canada	Cross-sectional	15	7–10 y M 8.13 y	Non-specified	Piezo SC-Step pedometer/Piezo SC-Step pedometer + questionnaire	Parent-child total PA: *P* = 0.597 (−)	Serious
Jago et al. [49] - 2017 - United Kingdom	Longitudinal	1223	5–6 and 8–9 y M 6 ± 0.4 y M 9 ± 0.4 y	Children and at least one parent	ActiGraph wGT3X-BT accelerometer/ ActiGraph wGT3X-BT accelerometer	Mother-daughter MVPA: *P* = 0.29 (+) Mother-son MVPA: *P* = 0.05 (+) Father-daughter MVPA: *P* = 0.05 (+) Father-son MVPA: *P* = 0.31 (+)	Moderate
Walsh et al. [50] - 2017 - Australia	Cross-sectional	140	20 months-5 y M 20 months M 3.5 y M 5 y	First born child and father	ActiGraph GT1M accelerometer/ The Active Australia Survey	Father-child MVPA: *P* = 0.02 (−) Father-child LPA: *P* = 0.181 (−)	Moderate
Dlugonski et al. [51] - 2017 - USA	Cross-sectional	17	1–5 y M 3.4 ± 1.4 y	Child and mother	ActiGraph GT3x-BT accelerometer/ ActiGraph GT3x-BT accelerometer	Mother-child MVPA: *P* = 0.78 (+)	Serious
Barkin et al. [52] - 2017 - USA	Cross-sectional	1003	2–5 y M 3.9 ± 0.9 y	Child and parent	ActiGraph GT3X or GT3X+ accelerometer/ ActiGraph GT3X or GT3X+ accelerometer	Parent-child LPA: P = 0.00 (+)	Moderate
Hnatiuk et al. [53] - 2017 - Australia	Cross-sectional	206	1–3 y M 2.7 ± 0.85 y	Children and mother	ActiGraph GT3X accelerometer/ ActiGraph GT3X accelerometer	Mother-child total PA: *P* = 0.17 (+)	Moderate
Maltby et al. [54] - 2018 - Canada	Cross-sectional	24	2.5–5 y M 3.76 ± 0.89 y	Child and mother	Actical accelerometer/ Actical accelerometer	Mother-child LPA: *P* = 0.085 (−) Mother-child MVPA: *P* = 0.105 (+)	Moderate
Xu et al. [55] - 2018 - China	Cross-sectional	247	5–6 y M 57.5 ± 5.2 months	Child and a legal guardian	ActiGraph GT3X accelerometer/ActiGraph GT3X accelerometer	Mother-child MVPA: *P* = 0.03 (+) Mother-child total PA: *P* = 0.20 (+) Father-child MVPA: *P* = 0.22 (+) Father-child total PA: *P* = 0.074 (+)	Moderate
Lee et al. [41] - 2018 - Canada	Cross-sectional	123	1–2 y M 1.6 ± 0.2 y	Children and parents	ActiGraph wGT3X-BT accelerometer/The Canadian Health Measures Survey	Parent-child MVPA: *P* = 0.295 (+)	Moderate

^a^*PA* Physical activity, ^b^*M* mean, ^c^*VPA* vigorous physical activity, ^d^*MVPA* moderate-to-vigorous physical activity, ^e^*LPA* light physical activity

### Quality assessment

For quality assessment of the selected studies, we developed a tool by modifying ROBINS-I [56] to fit our study question. The ROBINS-I items chosen were those which best captured the internal validity of the included articles (Additional file 3) [56]. The quality assessment tool then covered three domains: selection bias, information bias, and risk of bias in selection of the reported results. Because we were unable to conduct conventional meta-analysis, we added a judgement of risk of a type 2 error to the quality assessment tool. We judged the risk of type 2 error on the basis of a power calculation made by one of the authors (AG). It showed that to achieve a power of 80% to detect a correlation coefficient of 0.2 between parent and child PA with an alpha of 5%, at least 200 child-parent dyads were necessary.

The rest of the quality assessment was based on an appraisal of individual aspects of a study’s design, conduct, and analyses. Two authors (TLP and LBM) used the quality assessment tool to extract data from the included studies and for assessment of study quality and evidence synthesis. Each of the three ROBINS-I domains: selection bias, information bias, and risk of bias in selection of the reported results were scored using the ROBINS-I categories: low, moderate, serious, or critical risk of bias or no information [56]. Information bias in relation to objectively measured PA were scored by one of the authors (JCB) with great expertise in this field. Judgement of overall risk of bias was done across bias domains guided by the ROBINS-I recommendation (Additional file 4) [56].

### Synthesis of results

The included studies were too heterogeneous for conducting meta-analysis, because effect estimates were provided in non-homogeneous measures such as correlation coefficients, odds ratios, and regression coefficients and with insufficient information to calculate a homogenous effect size across all studies. To assist the interpretation of the results across studies, we therefore produced albatross plots [57] for all studies combined and by age-group (preschool-aged versus school-aged children), sex of parents (maternal, paternal, unspecified), sex of the child (boy, girl, unspecified), methodology of assessment of parent PA (objective, self-report), and type of PA measure examined (MVPA, steps, total PA, light PA (LPA)). An albatross plot is a scatter plot of each individual study size (sample size) against *p*-values (two-sided) from the effect estimates. The plot provides the possibility to interpret contours of estimated standardized effects size (here standardized to correlation coefficients) for a given p-value and study size for each individual study and for the overall relationship across studies [57]. To obtain data for making these plots, we extracted *p*-values, sample size, and estimate of effect for each study. If studies did not report exact p-values, we estimated these based on the sample size and size of effect (e.g. Pearson’s correlation). Also, if a study included multiple p-values from analyses of different PA outcomes (e.g. weekend and weekday estimates), we combined these for calculation of the study’s p-value for the albatross plot using available formulas [58]. For the albatross plot for all studies combined and for those studies reporting on the parent-child relationship in multiple ways, we prioritized to include estimates of association from 1) analysis reporting on a measure of total PA (prioritized in the order: total volume, time in MVPA, other PA measures); 2) objective assessment over self-report of parental PA; and 3) estimates obtained from analysis of non-specific gender of parent and child if the study reported on parent-child relationship in PA in multiple combinations of parent-child dyads. Duncan et al. [35] reported on the parent-child relationship in sub-groups of ethnicity of the child, which were independent observations, and these were treated as separate data points (one for each ethnic group) in the plots.

## Results

A total of 4205 articles were identified through database searching (Fig. 1). After removing duplicates, 2276 papers remained. Eight additional papers were identified through screening of references in other reviews [8–12, 59–61]. After the screening of abstracts, 82 papers were selected for full text screening. In the full text screening, 43 studies were excluded either because they did not report associations between parent and child PA or did not use objective measures to assess child PA. Finally, 39 studies met the inclusion criteria for the current review (Fig. 1).

### Characteristics of the included studies

The characteristics of the included studies are presented in Table 1. The majority (*n* = 36) were published within the last 10 years (2009–2018). Half of the studies were conducted in the US or Canada (*n* = 19) [19, 20, 24, 25, 27, 29, 31, 35–37, 42, 43, 45, 47, 48, 51, 52, 54, 62], and the rest were carried out in Europe (*n* = 12) [13, 21, 23, 26, 28, 33, 34, 38, 44, 46, 49, 63], Australia (*n* = 6) [30, 32, 40, 50, 53, 64], New Zealand (*n* = 1) [22], or China (*n* = 1) [65]. The majority of the studies were cross-sectional (*n* = 31), while the rest had a longitudinal design. Reporting of sampling and recruitment were lacking or unclear in many of the papers, but it seemed that recruitment of participants in almost half of the studies were done for other research purposes than examination of PA (e.g. studies on obesity in children) [28]. Many studies used non-probability sampling and recruited via e.g. fliers or newspapers.

The age of the children in the included studies ranged from 3.5 months to 18 years. Twenty-five studies examined school-aged children (7–18 years) [13, 19–21, 23–26, 29, 32, 34–37, 39, 42–46, 48, 49, 55] and fourteen focused on preschool-aged children (0–6 years) [22, 27, 28, 30, 31, 38, 40, 41, 47, 50–52, 54, 66], of which two studies [30, 41] were on infants/toddlers aged 3.5 months–20 months.

Sample size of parent-child dyads ranged from 15 to 1328 (mean = 319 dyads, median = 227 dyads). Inclusion criteria for 15 of the studies were cohabiting mothers and fathers and participation of both parents [13, 19, 21–23, 25, 28, 32, 34, 38, 39, 41, 42, 44, 66]. Details on each included paper regarding the family structure of parents and children are included in Table 1.

### PA measurement and outcomes

Thirty-one studies applied accelerometers to assess child PA [13, 19, 20, 22–25, 27, 28, 30, 31, 33, 35–38, 40, 41, 43–47, 49–55, 66]. Of these, twenty-one studies used accelerometers also for parent PA [13, 19, 22, 25, 27, 28, 31, 33, 36, 37, 43–47, 49, 51, 52–55], while ten of the studies used questionnaires [23, 24, 35, 38, 40, 41, 50, 66]. Eight studies used pedometers for both children and parents [21, 26, 29, 32, 34, 39, 42, 48]. Of these, three combined pedometers and questionnaires to assess parent and child PA [21, 42, 48].

The majority of the included studies (*n* = 32) used an average of total PA time as one outcome in the statistical analyses [13, 19, 20, 22–24, 26–30, 32–39, 41–44, 46, 48–50, 52, 54, 55, 64], while 18 reported MVPA and a few used LPA. The nine studies using pedometers reported steps per day.

Four studies looked at both the total day and specific time intervals of the day [21, 31, 40, 45], while two studies only examined specific time intervals of the day [25, 53]. Two studies analysed the time where parent and child were engaging in PA together [47, 51]. Fourteen studies distinguished between PA time of weekdays versus weekend days [25, 26, 28, 30, 33, 34, 36, 39, 43, 45, 51, 52, 55]. Because of a very limited number of homogenous estimates of relationship in PA between parents and children across studies in relation to time of the day and time of the week, analyses were not meaningful.

Reporting of the data reduction from raw accelerometer data to processed PA outcomes was either absent or revealed a non-homogeneous use of thresholds (e.g. five different thresholds for child MVPA), epoch lengths, non-wear-time, and definition of a “valid day” (Additional file 4).

### Associations between parent and child PA

We made albatross plots across studies (data points) to visually provide the estimated sizes of the relationships standardized to a correlation coefficient and to estimate the average magnitude and range of correlations. In the thirty-nine studies, 41 analyses provided some measures of the association between parent and child PA. The descriptive data used for the plots and the results are presented in Table 2.

**Table 2:** Descriptive data and results of associations of parent-child PA^a^ from albatross plots

	N studies	N data points	N total participants	Average magnitude of correlation (range)
All studies identified	39	41	11,553	0.13 (−0.26–0.40)
Low or moderate risk of bias	19	19	6735	0.15 (−0.19–0.40
Serious or critical risk of bias	20	22	5140	0.11 (−0.26–0.36)
Parent-son	9	9	3344	0.12 (−0.11–0.29)
Parent-daughter	10	12	3886	0.12 (−0.12–0.28)
Mother-child	18	18	5051	0.13 (−0.26–0.34)
Father-child	15	15	4069	0.12 (−0.19–0.36)
Total PA	10	10	2394	0.11 (−0.14–0.30)
Moderate to vigorous PA	20	22	6604	0.13 (−0.19–0.40)
Light PA	6	6	2001	0.03 (−0.33–0.23)
Steps	7	7	1584	0.18 (0.04–0.31)
Objectively assessed parent PA	29	29	9258	0.16 (−0.14–0.40)
Self-reported parent PA	10	12	2526	0.04 (−0.26–0.28)

^a^*PA* Physical activity

**Fig. 2:**
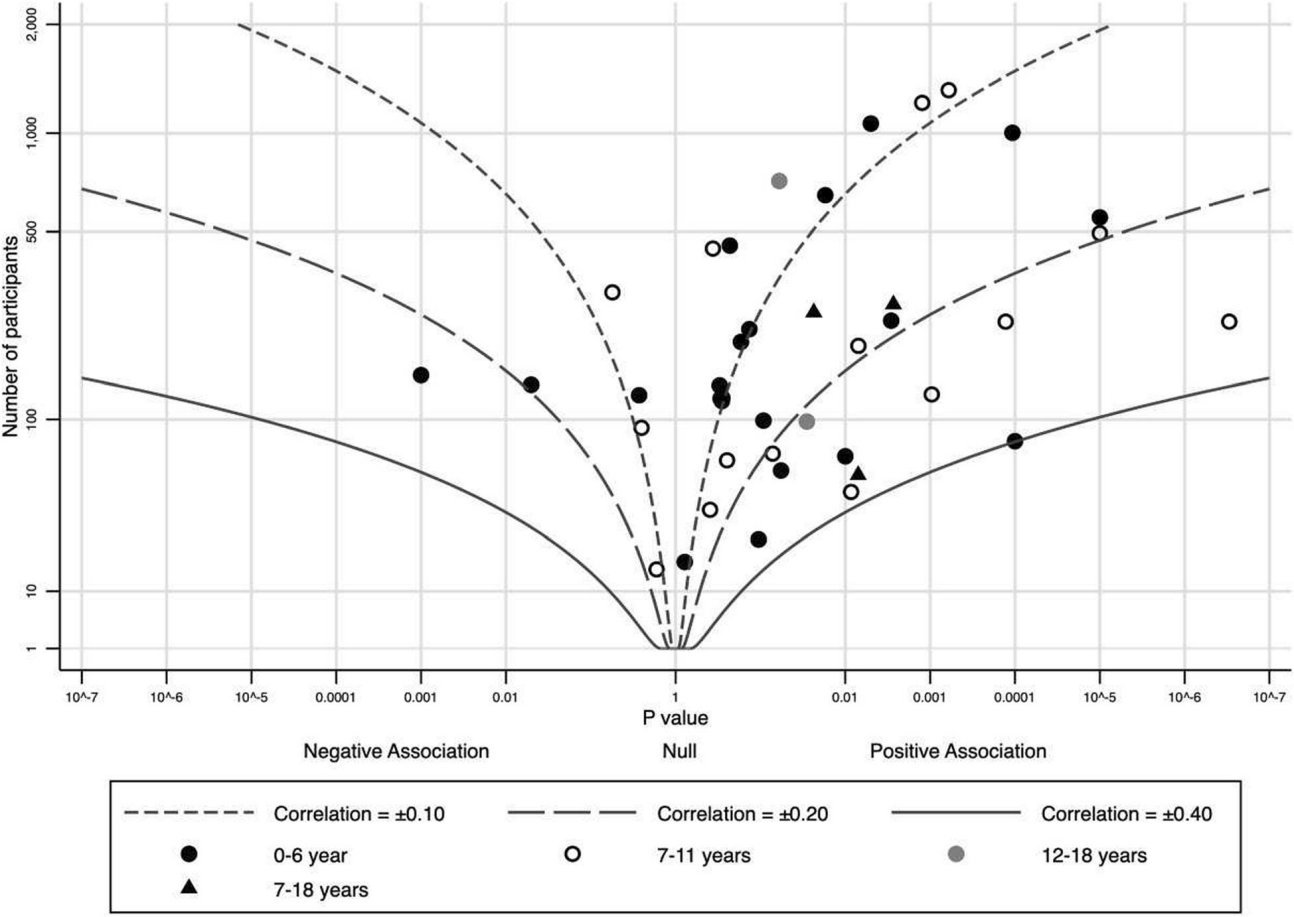
The overall albatross plot for the 39 included studies

The overall albatross plot that combined the 39 studies (Fig. 2) revealed that the clear majority, i.e. 35 of 41 data points, observed a positive relationship between parent and child PA mostly with a correlation coefficient between 0.10–0.20. The plot suggested an average magnitude of correlation across studies to be around 0.13, and the overall impression was that this was fairly similar across age-groups. When dividing the studies into two groups scoring low or moderate risk of bias or serious or critical risk of bias, the plot suggested an average magnitude of 0.15 and 0.11, respectively (Additional file 5).

Also when using data on different methods (Additional file 6), different intensities of PA (Additional files 7, 8, 9 and 10), and different gender combinations (Additional files 11, 12, 13 and 14), most of the albatross plots showed a positive relationship between parent and child PA. However, objective measures of parent PA suggested a higher average magnitude of correlation (0.16) than studies using self-report to measure parental PA (0.04) (Additional file 6).

PA outcomes varied across studies, but MVPA was most frequently used (*n* = 20). The albatross plot suggested an average magnitude of correlation across studies to be around 0.13, with the strongest association for the age-group 0–6 years (Additional file 7). Only studies in preschool-aged children (*n* = 6) looked at the relationship between LPA of parents and children. The plot gave an average magnitude of correlation across studies as low as 0.03 (Additional file 8). Ten studies used total PA as outcome (Additional file 9). Seven of these were conducted in generally small samples of preschoolers. The average magnitude of correlation across studies were 0.11. Two out of the three studies conducted in school-aged children showed a negative association between the total PA of parents and children. Seven studies used pedometer derived steps as PA outcome (Additional file 10). They were conducted in school-aged children and observed a positive association between parent and child. The albatross plot suggested an average magnitude of correlation across studies to be around 0.18.

Gender specific sub-group analyses showed a positive relation between parent and child PA regardless of gender and type of child PA. For father-child PA (*n* = 15), the plot showed an average magnitude of correlation to be around 0.12 (Additional file 11), while the average magnitude of correlation between mother and child PA was 0.13 (Additional file 12). The albatross plots both for parent-son and parent-daughter PA showed an average magnitude of correlation of around 0.12 (Additional files 13 and 14).

### Age of child

Overall, studies in school-aged children had larger sample sizes than studies in preschool-aged children. Most of the studies on preschool-aged children (0–6 years) looked at mother-child dyads. They reported on MVPA, LPA, or total PA and found a positive but weak association between parent and child PA. There was a tendency that studies with small sample sizes (*n* < 200 parent-child dyads) reported the strongest associations.

Among the studies examining the relationship between parents’ and school-aged children’s (7–18 years) PA, the majority were conducted in the youngest age-group (7–11 years). The studies in school-aged children mainly reported on steps or MVPA and different gender combinations (e.g. father-child, mother-child, or parent-daughter) and reported a weak association.

### Quality assessment

The overall assessment scores for the quality of the 39 studies included in the present review are presented in Table 1. Only one study [28] was judged as having low risk of bias while eighteen [13, 22, 27, 31, 36, 38, 40, 41, 43, 45, 46, 49, 50, 52, 54, 55, 67, 68] were considered as having moderate risk of bias. Fifteen studies [19, 21, 24–26, 29, 30, 32, 34, 39, 42, 44, 48, 51, 66] were categorized as having serious risk of bias, whereas five studies [20, 23, 35, 37, 40] were judged as having critical risk of bias. The nine studies measuring child PA using pedometers were assessed as having serious or critical risk of bias, whereas the majority (*n* = 19) of the accelerometer studies had moderate risk of bias. Due to small sample size, i.e. 15–200 parent-child dyads, twenty-one studies were assessed as having risk of type 2 error [13, 19–23, 25, 27–30, 32, 36, 40, 41, 43, 48, 50, 51, 54, 68].

Amongst the three domains covered by the quality assessment tool, especially the domain on information bias was judged to have moderate to serious risk of bias due to poor transparency in the description of reduction of raw accelerometer data and lack of information about the parent-child dyad by biology and gender. In the selection bias domain, ten studies [20, 23–25, 32, 35, 37, 44, 51, 66] were judged to have serious to critical risk of bias because sampling and recruitment procedures did not reflect the target population (Additional file 4).

## Discussion

The main purpose of this review was to systematically summarize the current research evidence on the relationship between parent and child PA. The clear majority of the 39 included studies observed a positive relationship between parent and child PA regardless of the age of the child, the gender of the parent-child dyad, and the PA outcome. The size of the estimated positive relationship was considered weak in most studies with a correlation between parent and child PA between 0.10 and 0.20. Generally, the quality assessment of the included studies indicated that this field of research deserves better quality in all aspects of methodology to decrease the risk of bias.

The systematic review provides several key points on the relationship between parent and child PA. First, we found little evidence across studies to suggest a gender-differentiated size of resemblance in PA between parents and their children. This could indicate that fathers and mother’s modelling of PA behaviour is similar or that the choice of activity-related context in which to interact with their children tend to be alike. Second, although some previous studies have suggested that the association between the time that parents and children spend engaged in PA are stronger in young- and middle childhood compared to adolescence [12], we could not find support for this when comparing estimates of correlation across studies with different age-groups. Considering that adolescence is considered a period with greater peer influence this is somewhat surprising, yet, further studies directly comparing the effect of child age is necessary to conclude with more confidence. Thirdly, considering the tendency for a positive but weak relationship between parent and child PA across studies, strategies to promote child PA by modeling the behaviour by parents may therefore only have limited effect. Despite a weak relationship, parents may still play important roles in influencing their children’s PA by encouragement and support (i.e. support organized sport participation). Many factors are assumed to influence child PA, such as biological, psychological, social, environmental, policy-related, and global components. As part of this, family is believed to play a role for the PA of family members. However, in recognition of the complex and multidimensional nature of PA behaviour, the parental influence may be relatively weak. Finally, an important finding in our systematic review was that the relationship between parent and child PA was stronger in studies judged to have low or moderate risk of bias compared to serious or critical risk of bias, and in studies using objectively measures to assess parental PA compared to studies using subjective measures. These methodological factors are discussed further below.

### Measurement and data reduction

Our sub-group analyses indicated that methodological differences between studies explain some of the heterogeneity of the results of the present review. One of our inclusion criteria was the use of objective measurement of child PA. The use of objective methods to asses PA has substantially increased the opportunity to obtain measurements of different kinds of PA in regard to the pattern, frequency, intensity, duration, and volume of children’s PA. Further, when studies in children have used objective measures of PA, strong associations with health outcomes (e.g. adiposity and blood pressure) have been reported [69]. In adults, self-report instruments such as questionnaires have shown reasonable validity and reliability for determining intensity, type, amount, and bout duration of PA [70] and was therefore accepted as method for measurement of PA in parents.

Although objective measurement tools are more accurate in the quantification of PA than self-reporting methods [70], there are still several methodological challenges in using e.g. accelerometers to assess parent and child PA. In the included studies, five different thresholds were used for the assessment of time spent in MVPA, which clearly illustrates the lack of consensus in this field [71]. Seven studies failed to report the thresholds used. Some calibration studies recommend age-specific cut point while others recommend the use of the same cut point for all age-groups [72–75]. The use of cut points is a simple method for estimating time spent in different intensity domains, however, it could add substantial misclassification among the individual intensity domains [74]. The cut points used for identifying MVPA in the included studies ranged widely from 1672 to 3200 counts per minute. Thus, the time spent in MVPA with the cut point provided with one study [72] could be identified as LPA with the cut-point provided with another study [76]. Some of the studies used the same threshold for children and parents [28, 31] while across studies, the same cut point for MVPA was used in very young children (3.5–18.7 months) and older children (5–18 years) [29, 30]. Using the same threshold across age-groups may not result in meaningful differences in MVPA and may influence the size of the estimated parent/child PA relationship. The poor parent/child relationship identified with LPA as compared with MVPA warrants further investigation and highlights the possibility that the choice of cut points for LPA and MVPA could explain differences between studies.

The optimal selection of epoch length is important for the accurate assessment of time spent in MVPA [77, 78]. The studies included in this review used an epoch length ranging from 5 to 60 s with a majority of studies using the same epoch length for both children and parents. A review by Migueles et al. [71] discussed the use of different epoch length for the assessment of PA in children. The natural PA behaviour of younger children is often frequent bursts of PA for short duration, hence a shorter epoch length might be more appropriate and may prevent intermittent MVPA from being misclassified as LPA [79]. However, it is not only the sporadic nature of children’s activity but also the intermittent character of adult PA that makes epoch length a significant factor on the interpretation of accelerometer data [80].

The participants enrolled in the majority of the studies included in this review were instructed to remove the activity monitor during water activities and during sleep. Dealing with periods of not wearing the activity monitor is important for accurate assessment of PA [71, 78, 81, 82]. This has to be dealt with in the data reduction process and the definition of non-wear-time, in the use of methods to account for it, and in ensuring minimum amount of data (hours) required for a valid day. Nevertheless, the largest portion of the included studies did not report how they defined non-wear-time, the definition of a valid day, or how many days of valid days they required for inclusion into the analysis. As noted by Aadland et al. [83], stringent requirement apply for a valid day (hours) or a valid week (days) produces more reliable data, but could also lead to sample loss. Migueles et al. [71] recommended evaluating different criteria (sample size and reliability of the measure) in order to identify the best compromise. As suggested in a previous review by Trost et al. [73], a minimum of 4 days of valid data is recommended. Overall, these important choices in data reduction when using accelerometry may have contribute to differences in estimated size of relationship between the included studies.

In this review, half of the studies used questionnaires to estimate parental PA. Questionnaires may pose serious limitations because they provide less accurate estimates of PA levels than those obtained by objective methods [72, 84, 85]. In addition, because the degree of the relationship between objective and self-report measures of PA is only moderate [86], there may be a substantial amount of variance not shared by the two methods, and therefore objective and self-report measures are not interchangeable. As such, the methods used can also influence the associations of PA, thereby impairing the generalisation of the findings obtained with the use of one or the other method [87]. Our albatross plots of different methods (self-report versus objective) used for assessment of parental PA (Additional file 6) showed evidence of discordant sizes of relationship in PA between parent and child with higher correlations observed in studies using objective methods. This suggests that the choice of method to assess PA in both parents and children is important and should be the same for both groups.

### Selection bias

In the quality assessment domain ‘selection bias’, it was observed that some of the included studies recruited participants by the use of media advertisements or posters or included participants who already were volunteering in another research project. The lack of population-based recruitment and missing information on participating rate may have induced selection bias arising from selecting participants into the study, and the non-probability sampling could have produced bias in the associations between parent and child PA. Also, this could have lowered the external validity [88]. Nevertheless, the information needed to generalize findings and to potentially identify sources of bias were lacking in the majority of the included studies. This is unfortunate, because it influences the interpretation of the results, and makes it difficult to identify potential gaps for further investigation [88].

The transparency of the operationalisation and inclusion of parents varied across studies. Some studies included only biological parent-child dyads, while others used a broader understanding of the concept of family and included e.g. stepparents, grandparents, or foster parents. Several studies that recruited both fathers and mothers ended up including only one parent and one child – generally mother-child dyads – in their final analyses without a clear explanation for the choice. The lack of transparency in the operationalisation makes it difficult to compare studies.

The albatross plots regarding gender specific sub-group analyses on all types of child PA showed a positive relation between parent and child regardless of gender. However, the sample sizes were small and therefore, the analyses on the association between mother and child PA were at risk of type 2 bias.

### Strengths and limitations

A strength of this review is that we identified and retrieved studies using a broad search strategy that included five databases, which limits the possibility that we have missed published studies. However, we only included studies in English or Scandinavian languages and, research on this topic may have been published in other languages. Unfortunately, the retrieved data did not allow meta-analysis but instead we incorporated a quantitative graphical synthesis of the results using correlation as the standardized effect estimate. This provided us with the possibility to interpret the scatter of estimated correlation coefficients for a given *p*-value and study size for each individual study and for the overall relationship across studies. However, it is important to note that the estimated average magnitude of correlation was not based on a weighted average of the variance of each study estimate and the between study variance as in a random effect-meta analysis. This review examined associations and therefore, causal relationships could not be inferred.

#### Implications for research and conclusion

This review has identified areas needing further investigation. Although parental modelling has been well accepted as a possible mechanism for child engagement in PA [9], our main finding of a weak correlation between the level of parent and child PA suggests that we should develop a deeper understanding of associations and mechanisms. Additional dimensions worth examining may be activity types such as sitting, standing, running, and cycling. To broaden the understanding of PA in the familial context, taking siblings into consideration could also be fruitful.

The methodological weaknesses revealed in the present review should be tackled in future research. Therefore, we recommend greater transparency in every step of the research process including statement of methodological decisions regarding data reduction (e.g. thresholds for PA intensities, choice of epoch length, and criteria for the definition of a valid day) [79]. In addition, studies incorporating larger study samples may provide additional power to uncover potential associations not yet revealed.

This review showed that the clear majority of the included studies observed a weak positive relationship between parent and child PA regardless of age of the child, the gender of the parent-child dyad, and type of PA. Developing a better understanding of parental influence on child PA is important for identifying effective strategies for increasing children’s PA that could contribute to positive effects on health and well-being. Therefore, more research of high quality is needed in this field.

## Supplementary information

**Additional file 1.** PRISMA 2009 checklist.

**Additional file 2.** Search strategy.

**Additional file 3.** Modified ROBINS-I tool.

**Additional file 4.** Overall risk of bias judgement.

**Additional file 5.** All studies identified (albatross plot risk of bias).

**Additional file 6.** All studies identified (albatross plot method).

**Additional file 7.** Moderate and vigorous physical activity of child (albatross plot).

**Additional file 8.** Light physical activity of child (albatross plot).

**Additional file 9.** Total physical activity of child (albatross plot).

**Additional file 10.** Number of steps of child (albatross plot).

**Additional file 11.** Father-child relationship (all types of child physical activity) (albatross plot).

**Additional file 12.** Mother-child relationship (all types of child physical activity) (albatross plot).

**Additional file 13.** Parent-son relationship (all types of child physical activity) (albatross plot).

**Additional file 14.** Parent-daughter relationship (all types of child physical activity) (albatross plot).

## Data Availability

All data generated and analysed in this study are included in the present article and its supplementary information files.

## Abbreviations

LPA: Light physical activity
MVPA: Moderate-to-vigorous physical activity
PA: Physical activity
WHO: World Health Organization
