# Supplementary material for: Association between parent and child physical activity: a systematic review
Source: Int J Behav Nutr Phys Act. 2020 May 18;17:67. doi: 10.1186/s12966-020-00966-z (PMC7236180; doi:10.1186/s12966-020-00966-z)
Supplement: Supplementary file 2 — Additional file 2. Search strategy. [file 12966_2020_966_MOESM2_ESM.docx]

Additional file 2: Search strategy

| OR | AND, OR | AND, OR |
| --- | --- | --- |
| Family  Parents  Child  Adolescent  Child Behavior  Adolescen* (title/abstract)  Teen* (title/abstract)  “Young people” (title/abstract)  Family (title/abstract)  Parent* (title/abstract)  Child* (title/abstract) | "parental support" (title/abstract)  "parental style*"(title/abstract "parental influence"(title/abstract)  "parent and child*"(title/abstract) "child parent*"(title/abstract)  "parent child*"(title/abstract)  "family based"(title/abstract]  "mother and daughter"(title/abstract)  "mother and son"(title/abstract) "mother and child"(title/abstract) "child and mother"(title/abstract) "daughter and mother"(title/abstract)  "son and mother"(title/abstract) "father and son"(title/abstract)  "father and daughter"(title/abstract) "father and child"(title/abstract) "child and father"(title/abstract)  "son and father"(title/abstract) "daughter and father"(title/abstract) | "Motor Activity"  "Sports"  "Exercise"  "Physical Fitness" "Sedentary Lifestyle" "physical inactivity"(title/abstract) sedentary*(title/abstract)  "physical intens*"(title/abstract) "physical activ*"(title/abstract)]  "energyexpenditure"(title/abstract)  exercise*(title/abstract) sport*(title/abstract) |
