# Supplementary material for: Association between parent and child physical activity: a systematic review
Source: Int J Behav Nutr Phys Act. 2020 May 18;17:67. doi: 10.1186/s12966-020-00966-z (PMC7236180; doi:10.1186/s12966-020-00966-z)
Supplement: Supplementary file 3 — Additional file 3. Modified ROBINS-I tool. [file 12966_2020_966_MOESM3_ESM.docx]

Additional file 3: Modified ROBINS-I tool

ROBINS-I questions modified to this review.

“Is the reported effect estimate likely to be selected on basis of multiple outcome measurements within the outcome domain?” and “Is the reported effect estimate likely to be selected on basis of different subgroups?”. Other items where modified to fit this review, e.g. “Exposure measurement method listed in the study” was changed to “Is the self-reported PA measured validly and reliably by this method?”
