# Supplementary figures and images for: Association between parent and child physical activity: a systematic review
Source: Int J Behav Nutr Phys Act. 2020 May 18;17:67. doi: 10.1186/s12966-020-00966-z (PMC7236180; doi:10.1186/s12966-020-00966-z)

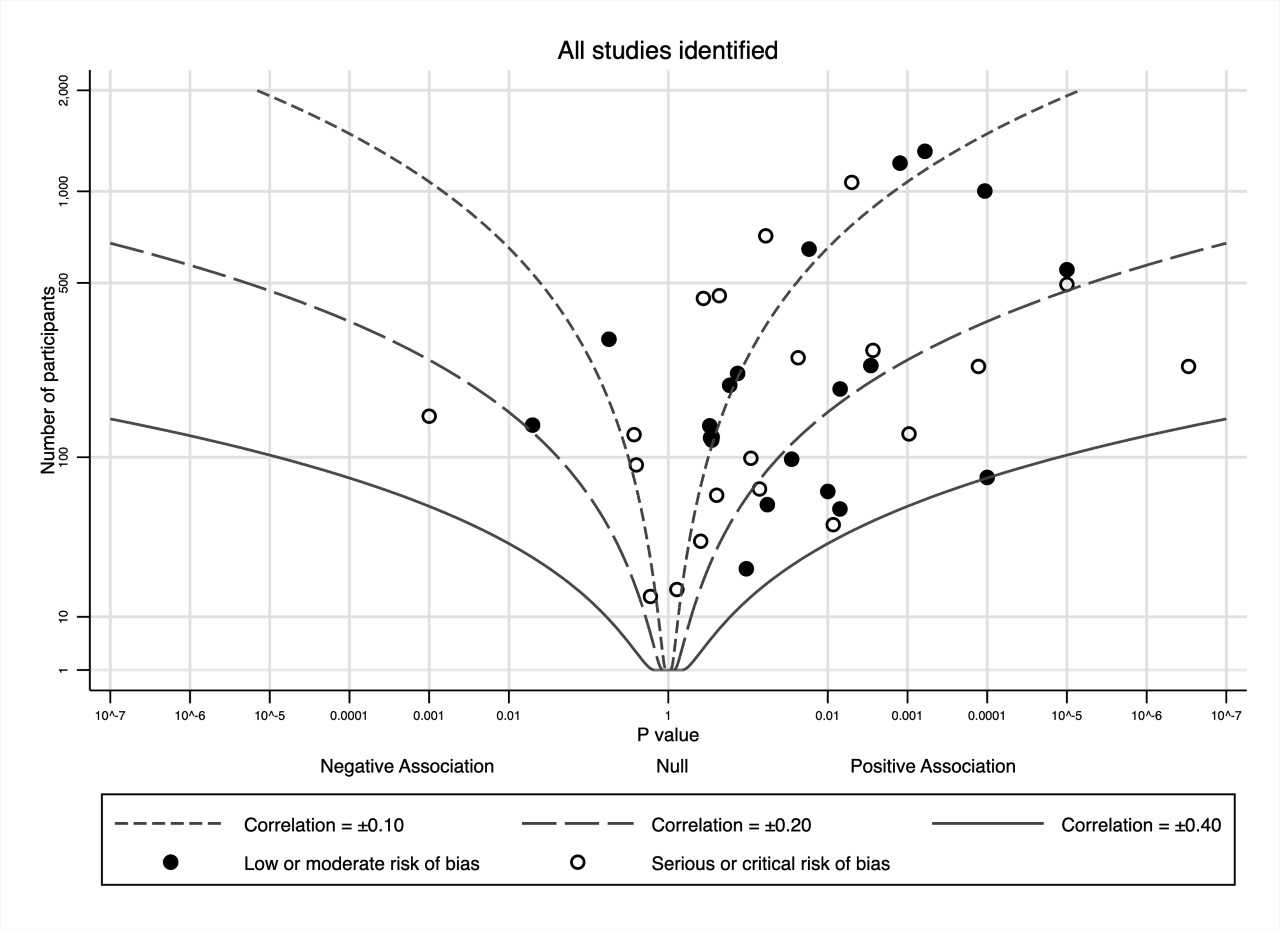

Supplement: Supplementary file 5 — Additional file 5. All studies identified (albatross plot risk of bias). [file 12966_2020_966_MOESM5_ESM.jpg]

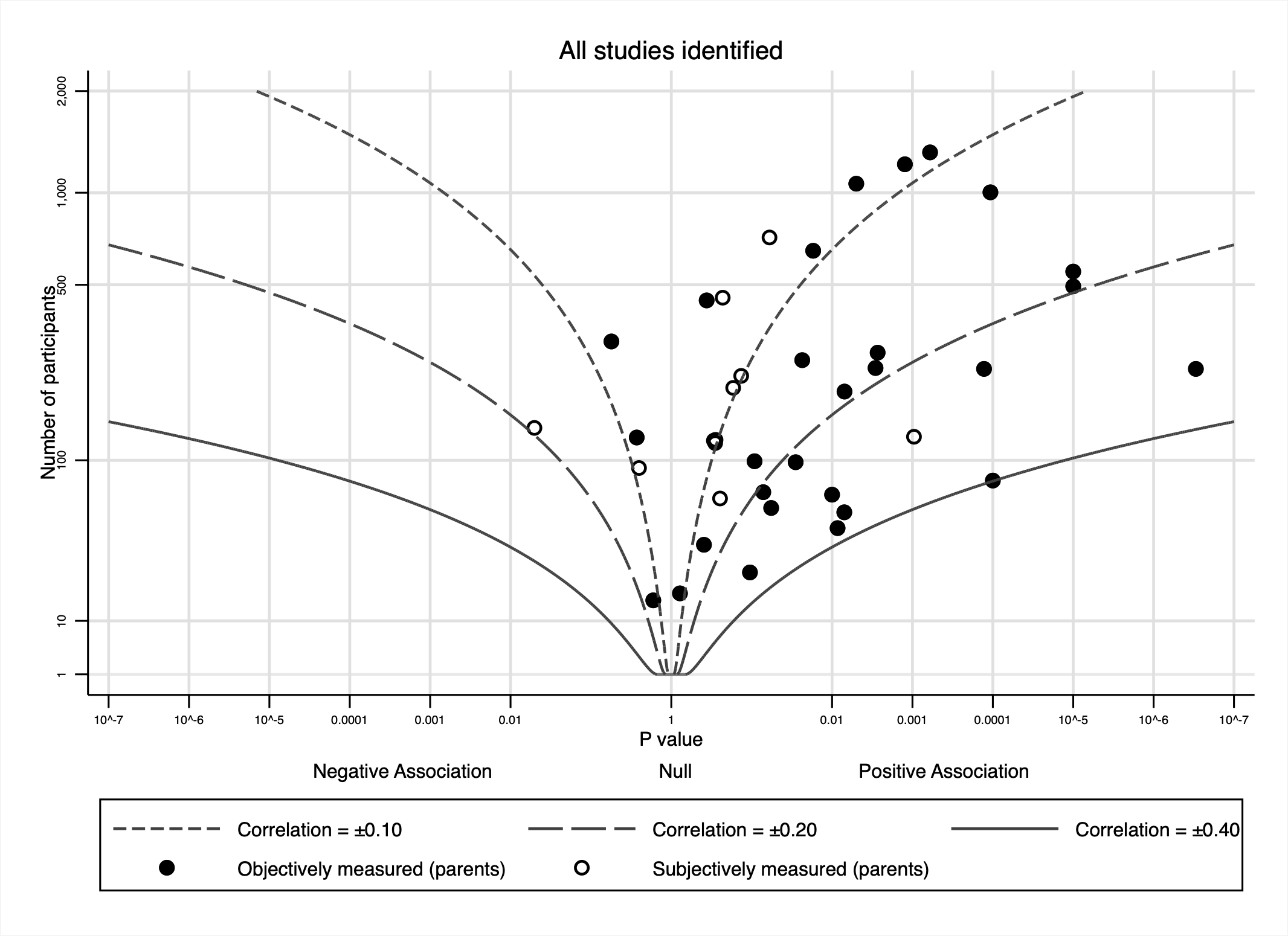

Supplement: Supplementary file 6 — Additional file 6. All studies identified (albatross plot method). [file 12966_2020_966_MOESM6_ESM.jpg]

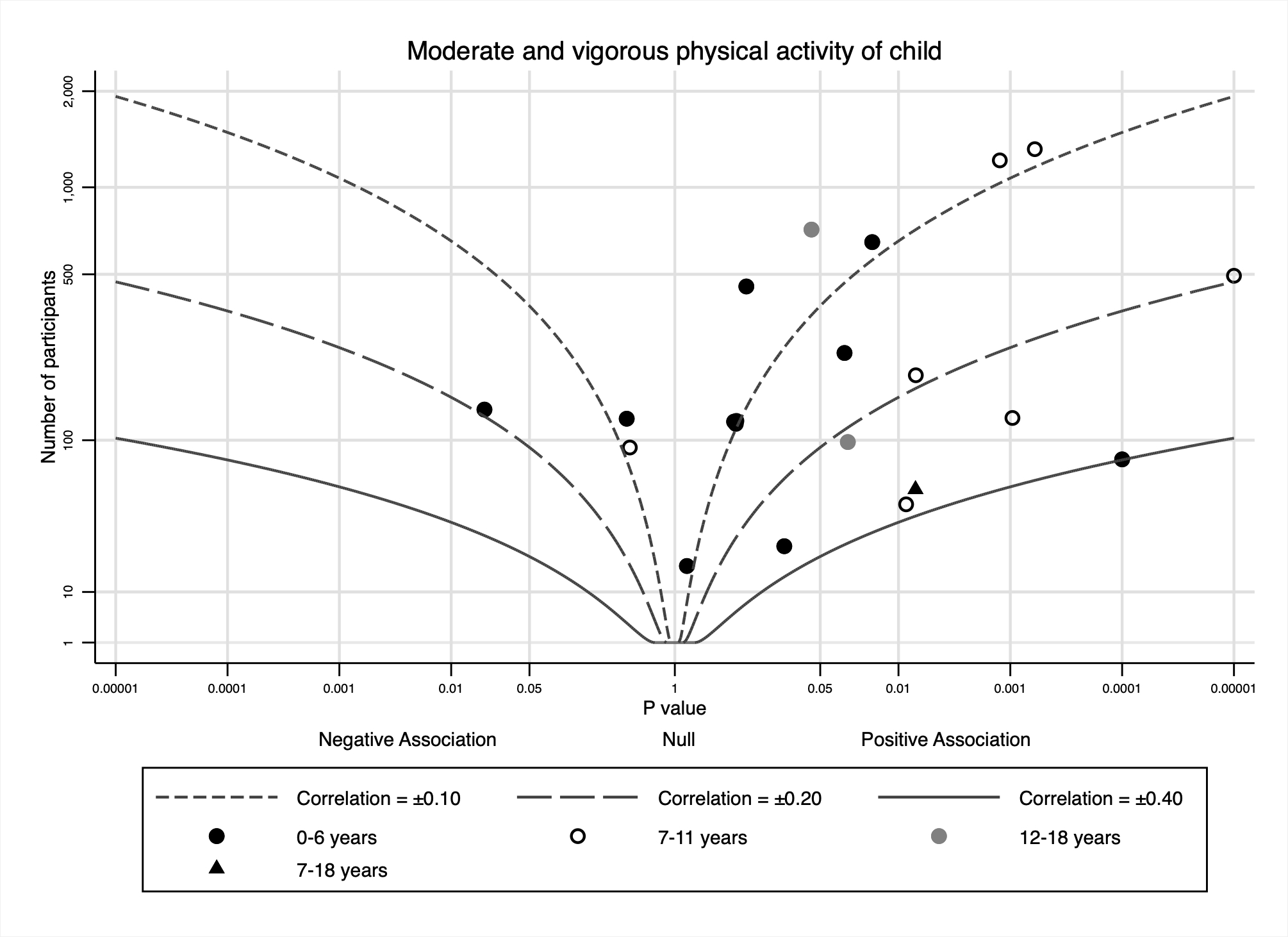

Supplement: Supplementary file 7 — Additional file 7. Moderate and vigorous physical activity of child (albatross plot). [file 12966_2020_966_MOESM7_ESM.jpg]

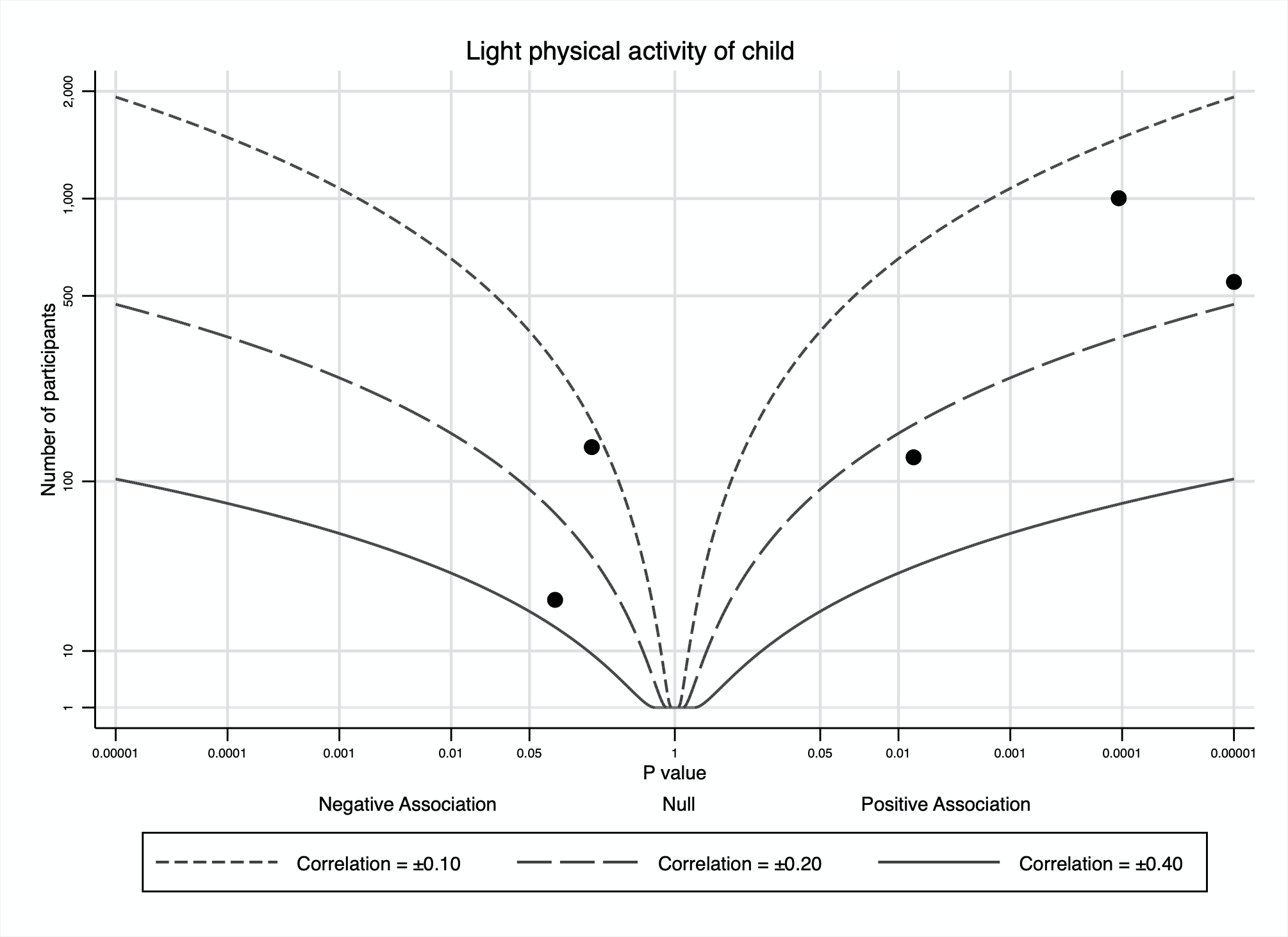

Supplement: Supplementary file 8 — Additional file 8. Light physical activity of child (albatross plot). [file 12966_2020_966_MOESM8_ESM.png]

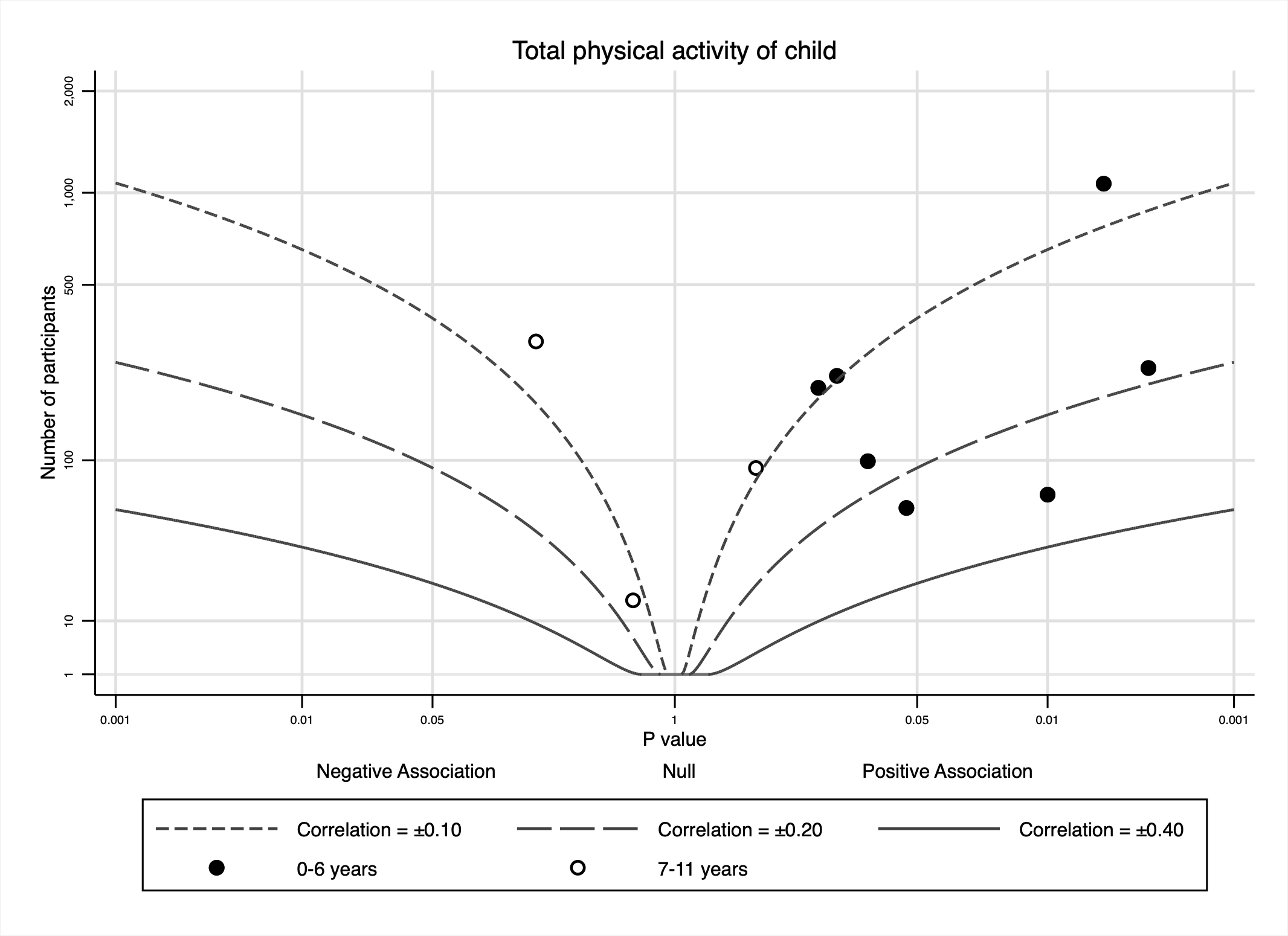

Supplement: Supplementary file 9 — Additional file 9. Total physical activity of child (albatross plot). [file 12966_2020_966_MOESM9_ESM.jpg]

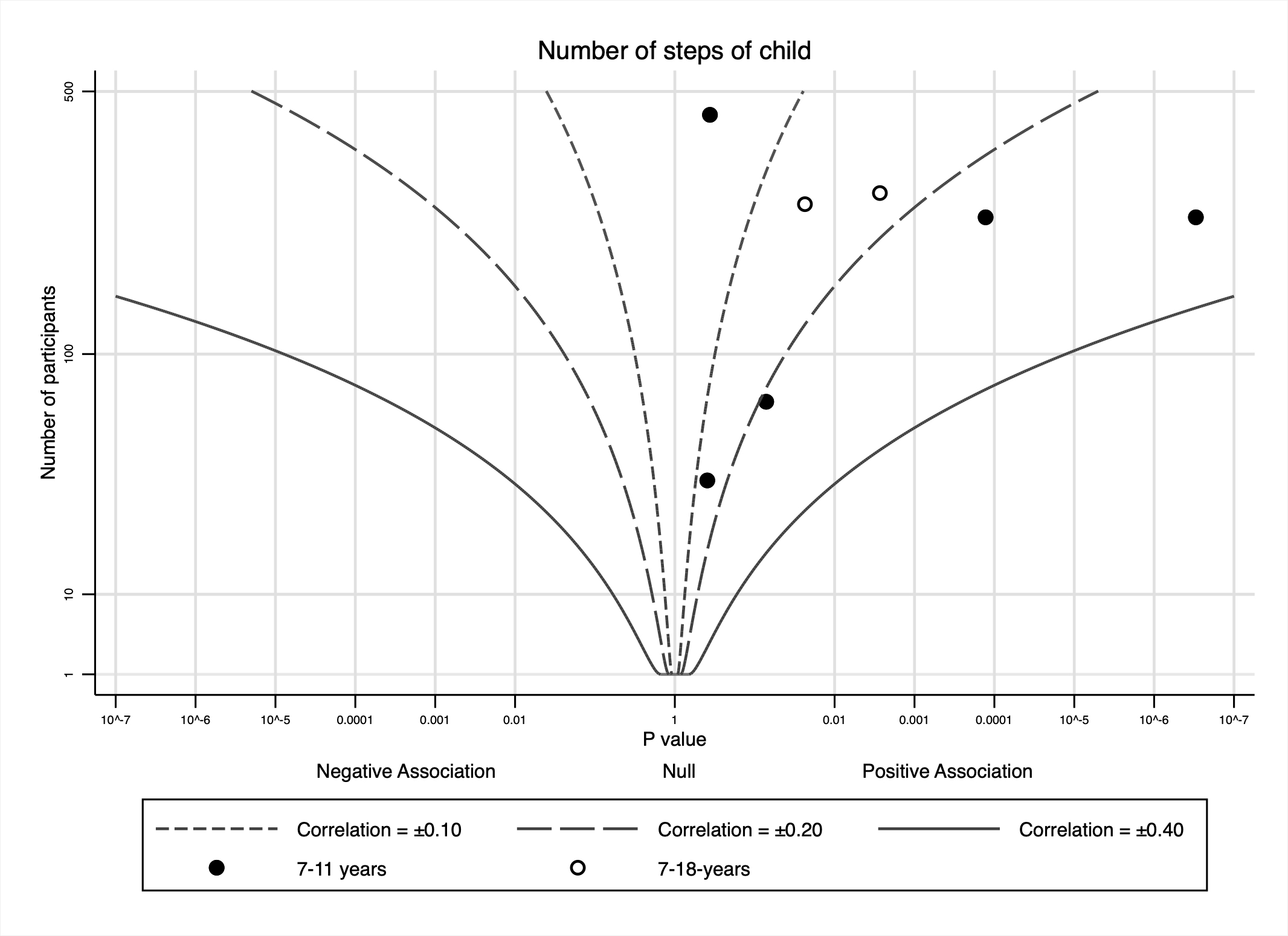

Supplement: Supplementary file 10 — Additional file 10. Number of steps of child (albatross plot). [file 12966_2020_966_MOESM10_ESM.jpg]

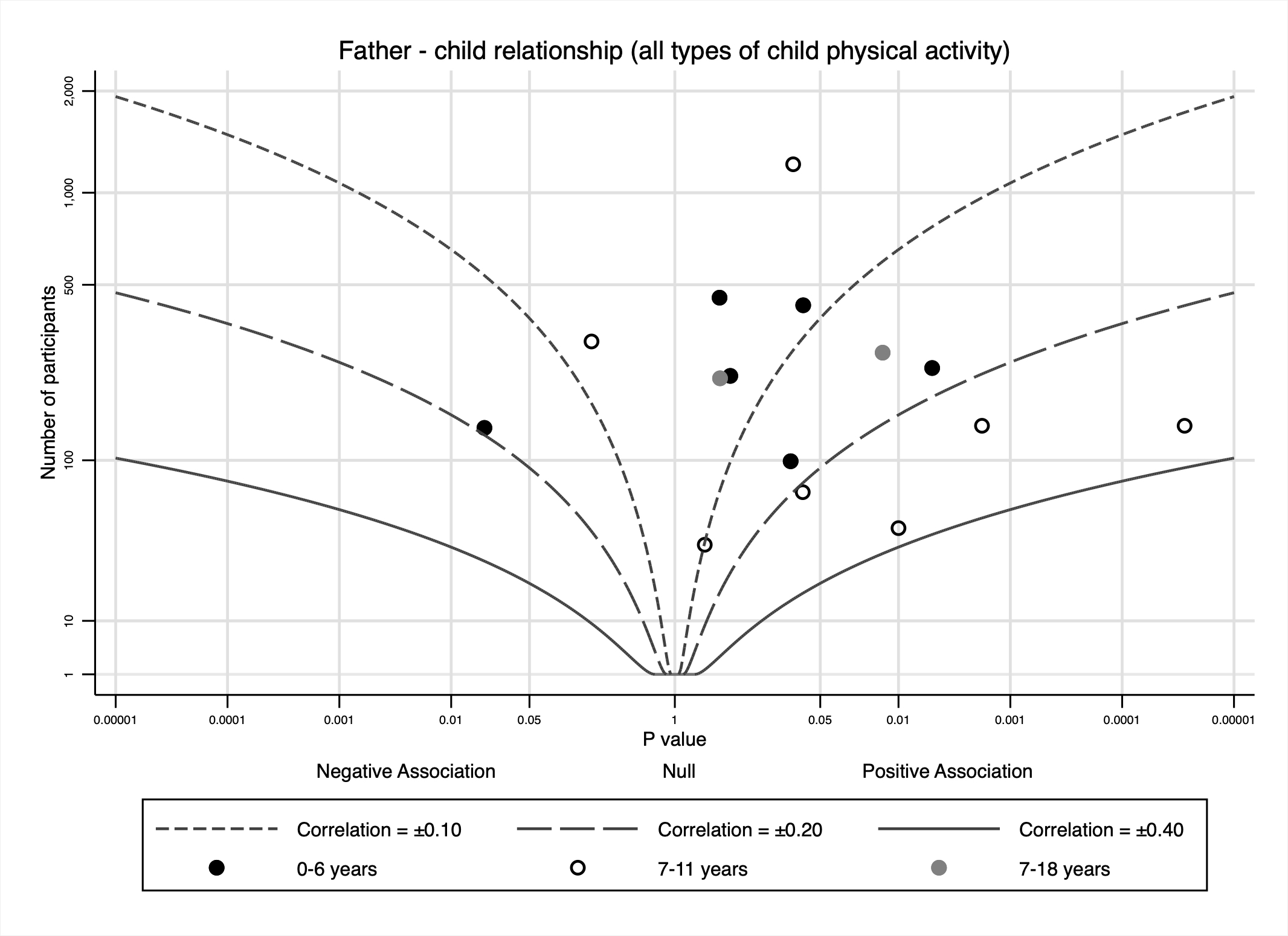

Supplement: Supplementary file 11 — Additional file 11. Father-child relationship (all types of child physical activity) (albatross plot). [file 12966_2020_966_MOESM11_ESM.jpg]

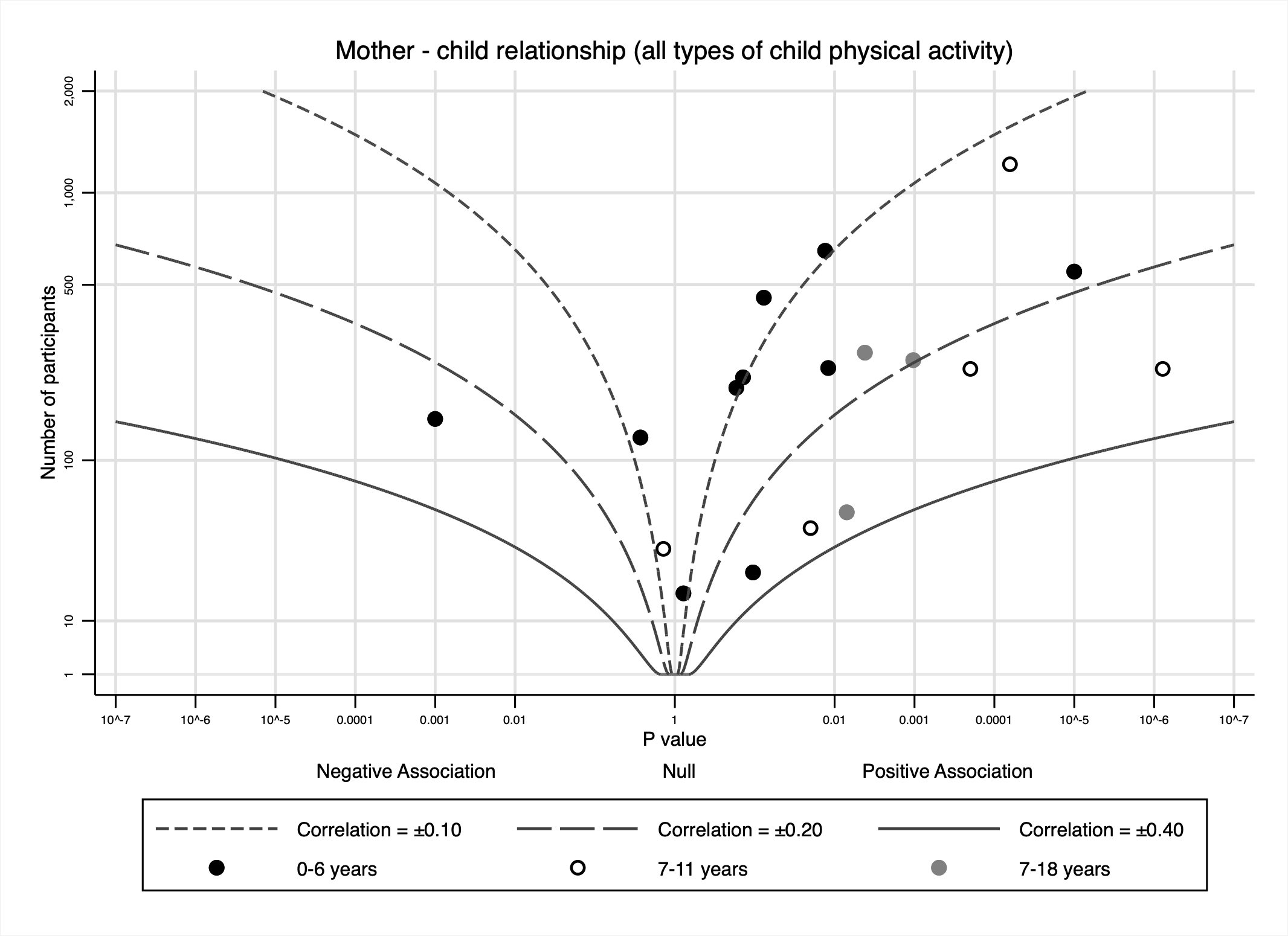

Supplement: Supplementary file 12 — Additional file 12. Mother-child relationship (all types of child physical activity) (albatross plot). [file 12966_2020_966_MOESM12_ESM.jpg]

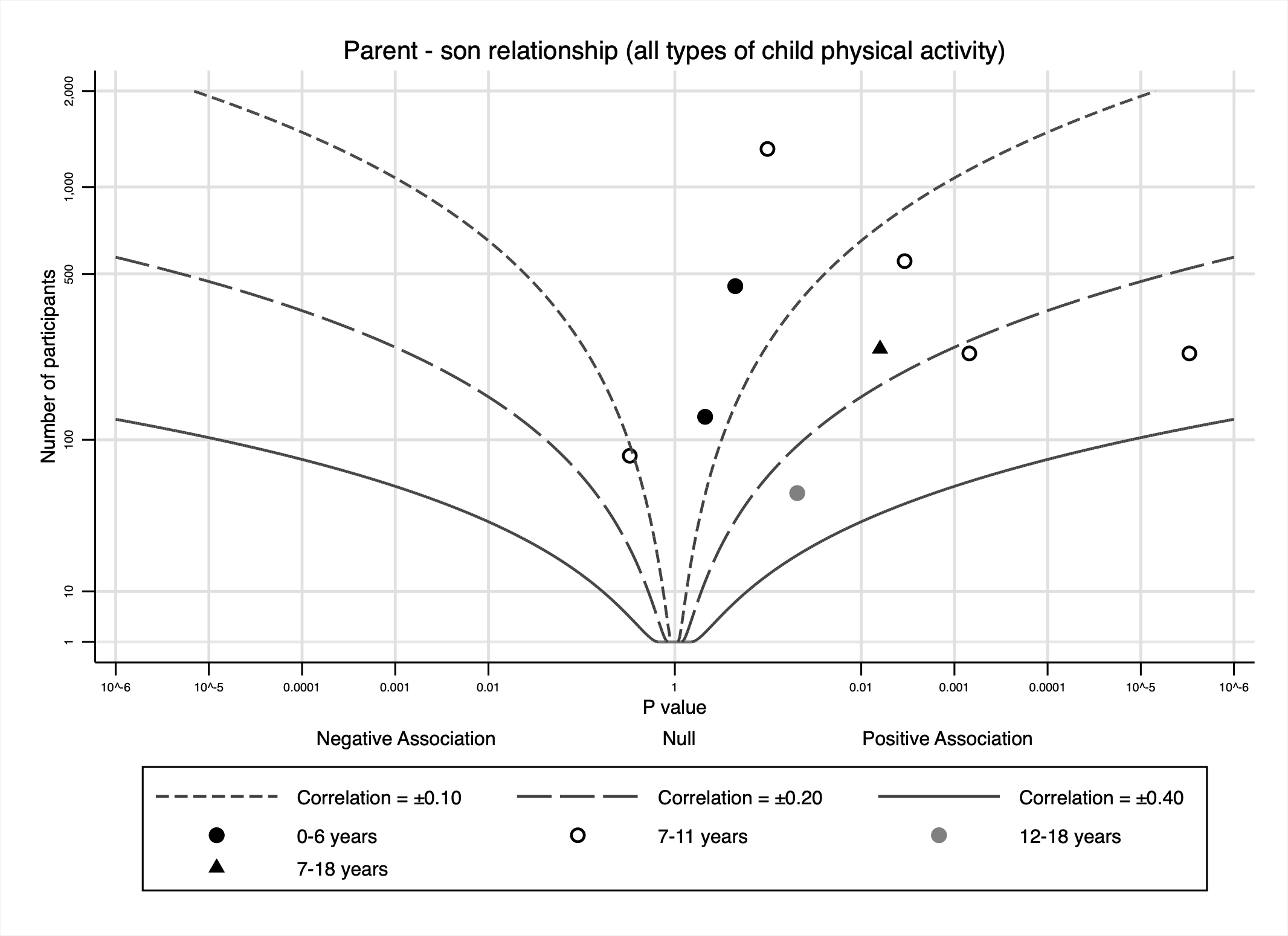

Supplement: Supplementary file 13 — Additional file 13. Parent-son relationship (all types of child physical activity) (albatross plot). [file 12966_2020_966_MOESM13_ESM.jpg]

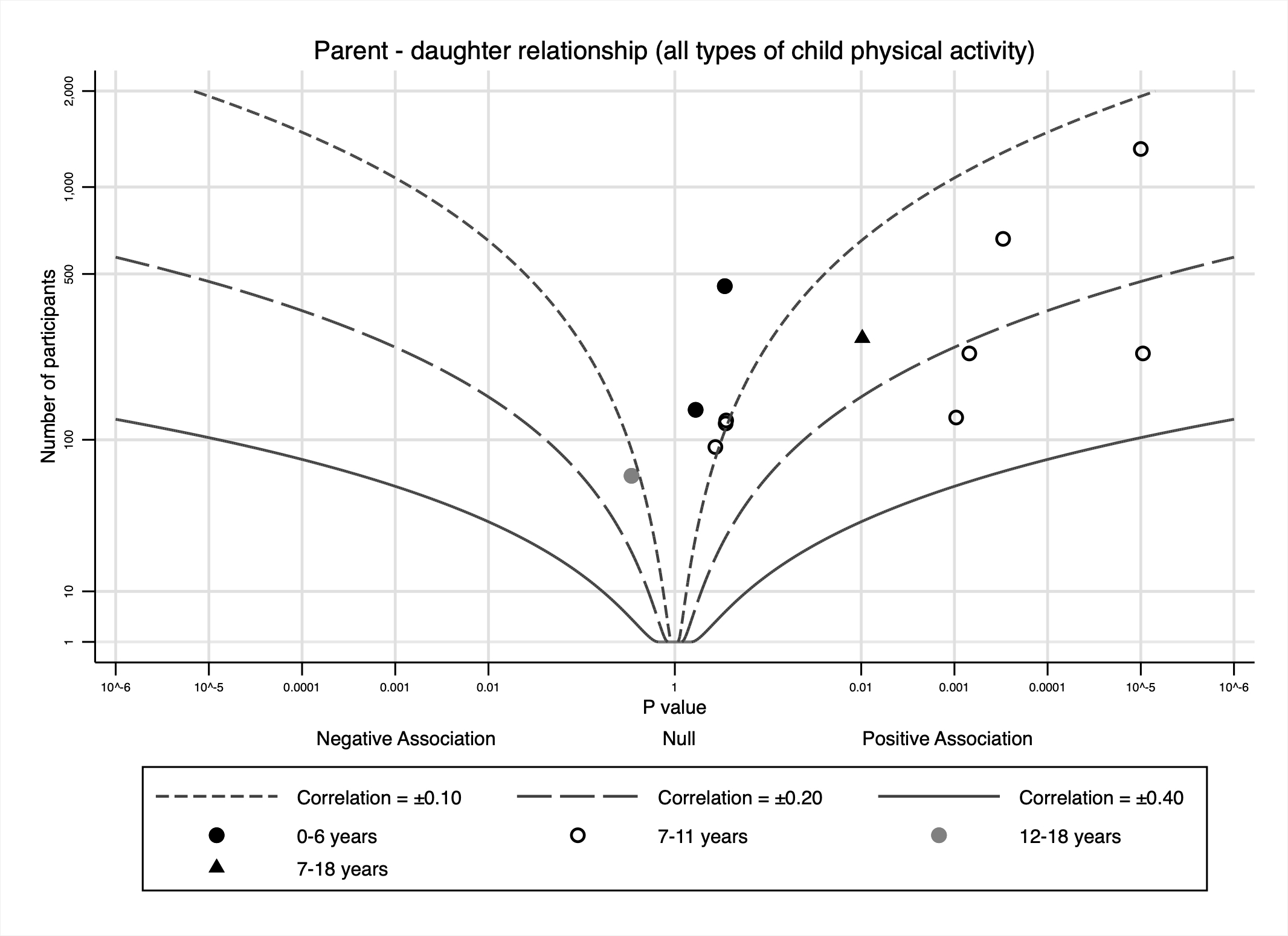

Supplement: Supplementary file 14 — Additional file 14. Parent-daughter relationship (all types of child physical activity) (albatross plot). [file 12966_2020_966_MOESM14_ESM.jpg]
